# Engineering yeast artificial core promoter with designated base motifs

**DOI:** 10.1186/s12934-020-01305-4

**Published:** 2020-02-18

**Authors:** Rui Liu, Lanqing Liu, Xia Li, Duo Liu, Yingjin Yuan

**Affiliations:** 1 Frontier Science Center for Synthetic Biology and Key Laboratory of Systems Bioengineering (Ministry of Education), School of Chemical Engineering and Technology, Tianjin University, Tianjin, 300350 People’s Republic of China; 2 SynBio Research Platform, Collaborative Innovation Center of Chemical Science and Engineering (Tianjin), Tianjin, 300072 People’s Republic of China

**Keywords:** Synthetic biology, Promoter, Core promoter, *Yarrowia lipolytica*, Lycopene, Carotene

## Abstract

**Background:**

Synthetic biology requires toolbox of promoters to finely tune gene expression levels for building up efficient cell factories. Yeast promoters owned variable core promoter regions between the TATA-box and transcriptional starting site (TSS) at the length mostly around 20–80 bases. This region allowed flexible design of artificial promoter but potentially demand special base motifs to maintain or enhance the promoter’s strength.

**Results:**

Here, we designed and screened the base motifs and tested the activities of yeast artificial core promoters. Different 30 bases of artificial sequences led to variable expression levels of CrtY enzyme which determined the lycopene–carotene compositions, represented in the colony-color spectrum of red–orange–yellow. The upstream sequences of two strong promoter P_EXP1_ and P_GPD_ and two starting strains with distinguishable lycopene production levels were utilized to characterize the promoter sequences. Different partition designs of T-rich or G/C-rich base motifs led to distinguishable colony-color distributions. Finally, we screened a champion promoter with a highest 5.5-fold enhancement of lycopene–carotene transformation. Another selected promoter generated a highest beta-carotene production as 7.4 mg/g DCW.

**Conclusions:**

This work offered an approach to redesign promoter with artificial sequences. We concluded that the core promoter region could be designated as 30 bases and different base motifs would enhance or weaken the promoter’s strength. Generally, more T-rich elements, higher %T and lower G/C percentage were beneficial to enhance the strength of artificial core promoter.

## Background

One of the characteristics of synthetic biology is de novo design and synthesis of biological functional parts and devices. Promoters are especially important for controlling the regulation-modes and strengths of gene expression, generating proper enzyme amounts to optimize cellular metabolism. Yeast is one of the most frequently used chassis in the research of synthetic biology, presenting excellent performance as cell factories to produce varied valuable biochemicals [1–3]. Appropriate promoter strengths drastically affected efficiency of heterologous synthetic pathways in yeast and consequent product compositions [4–7]. Recent works have established the modular architecture of yeast promoters, such as in baker’s yeast *Saccharomyces cerevisiae*, methylotrophic *Pichia pastoris* and oleaginous *Yarrowia lipolytica* [8–13]. Redden and Alper’s work is especially outstanding as they defined and proved a design mode of minimal yeast promoter that conserved high levels of expression with almost 80% reduction in size [8]. The minimal modular yeast promoter consists of several basic parts in order, namely, hybrid upstream activating sequence (UAS), neutral AT-rich spacer, TATA-box, N_30_ core promoter, and transcriptional starting site (TSS). This work offers a great chance for designing artificial promoters with more generalized sequences.

Among these modular parts, UAS, AT-rich spacer, TATA-box and TSS have distinctive conservative features as only limited number of natural or artificial sequences would preserve or enhance promoter’s strength but most engineered sequences drastically decrease promoter’s strength [6, 8–16]. By contrast, the core promoter sequence downstream of the TATA box influenced the gliding speed of RNA polymerase II before the mRNA was generated, also determining maximal promoter activity. This region allows more space of artificial design but also has its own limitedness, as the thoroughly randomized sequences will overwhelm effective sequence that enhance promoter’s strength. Portela and colleagues’ recent work made a remarkable chance to design universal mode of core promoter that could be used in different yeast species [17]. There still leaves a question whether this region can be replaced by a de novo designed artificial sequence.

**Fig. 1:**
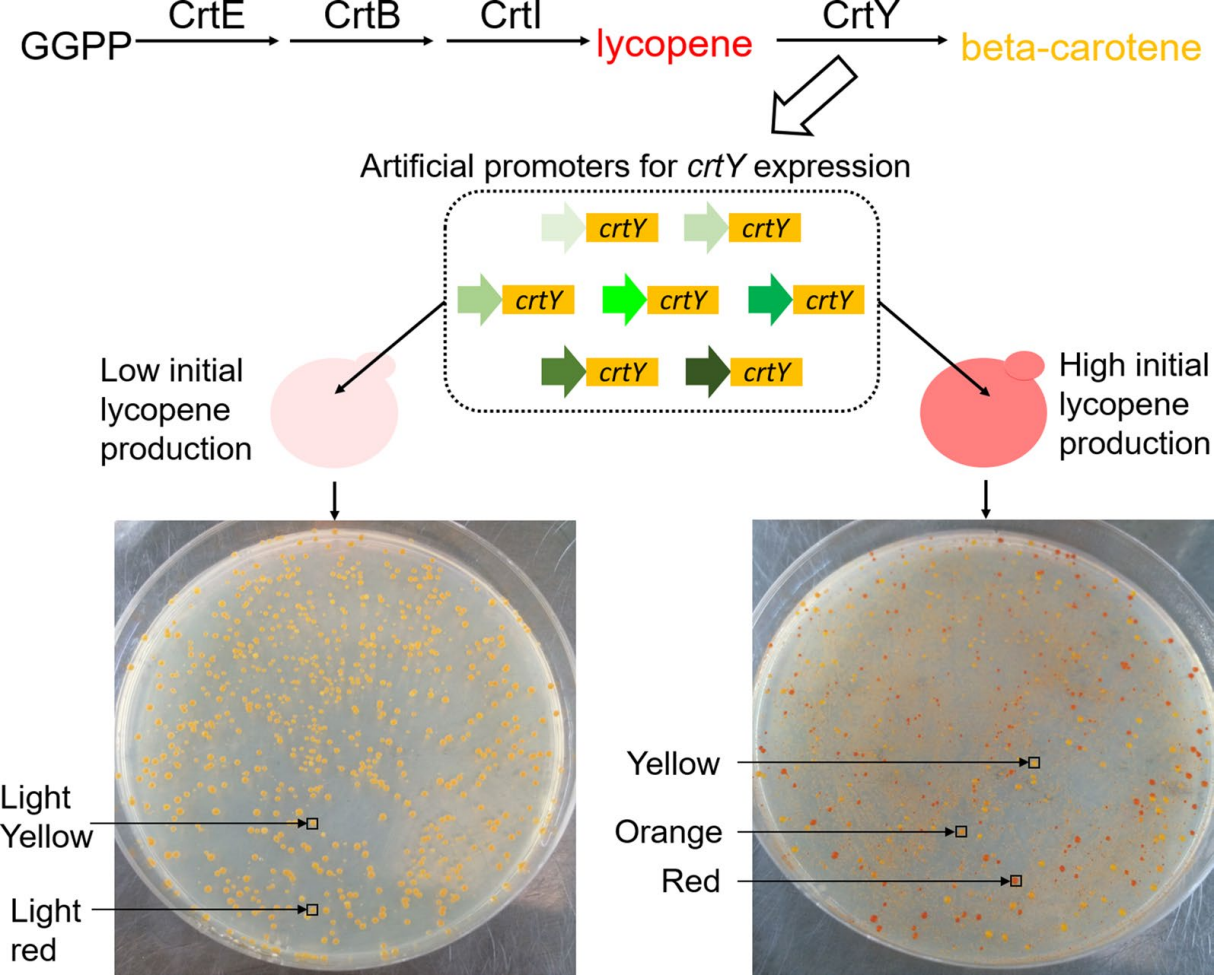
Concept of screening artificial core promoters assisted by yeast colony-color spectrum. The engineered artificial promoters changed the expression level of CrtY enzyme and tuned the lycopene–carotene composition, leading to variable yeast colony color distribution. In the strain with low lycopene production, we observed two colors as the original light red and new light yellow. In the higher-lycopene-production strain, we observed three colors as the original red and new orange and yellow

From another angle, we need this kind of de novo designed artificial promoters as barcodes to mark gene expression at genome level to research the genome rearrangement [18, 19]. The artificial barcodes of promoters will further assist the analysis of synthetic genomic evolution. Considering this point, we plan to construct a series of artificial core promoter sequences and characterize their performances to get some kind of rule. In the present study, we constructed artificial core promoters in *Y. lipolytica*, which was engineered for producing valuable chemicals as fatty acid derivatives, organic acids, terpenoids and sterols [20–22]. We chose an existing object that the artificial promoters might enhance the expression levels of CrtY enzyme to get higher beta-carotene production form the substrate lycopene (Fig. 1). We could rapidly screen desirable promoters contained in the yeast colonies with different colors of red–orange–yellow that presented different lycopene–carotene compositions (Fig. 1). The colonies with lycopene production of 1.2 mg/g DCW and much higher 25 mg/g would present highly differentiate color-spectrum. We designed two series of N_30_ core promoters. The first series were located downstream of a natural promoter P_EXP1_ and replaced the original 55 bases between TATA-box and TSS in P_EXP1_. The second series replaced the original region of 61 bases between TATA-box and the TSS in another natural promoter P_GPD_. Both promoters were commonly used and owned obviously stronger strengths than other promoters. Since N_30_ was large library, we here focused to test the influences of T-rich modules and G/C-rich modules on core promoter’s strength.

## Results and discussion

### Design and construction of artificial core promoter

In previous studies it was proved that the RNA polymerase II of *S. cerevisiae* performed a downstream scan of the core promoter region, searching for TSS sequence, which was distinct from metazoan transcription mode [23, 24]. We hypothesized that the corresponding region was also highly changeable in *Y. lipolytica*. Partially according to Redden and Alper’s work [8], we set 30 bases as the standard length of core promoter libraries. The 30-base sequence could be high-efficiently inserted into the assembled short DNA segments with our previous method [6]. Further, we could ligate these short DNA segments into special constructed vectors to get whole *crtY* transcriptional units. The details were shown in Additional file 1: Figure S1 and Table S1.

The 30 random bases of N (N = A/T/C/G) would lead to a very large library (4^30^ = 10^18^ possibilities) and could not be adequately characterized by transformed yeast colonies (500–1000 colonies per plate). Thus, we simplified the condition and divided the 30 bases into three parts of 10 bases and set each part with T-rich bases or G/C-rich bases (Table 1). Detailly, we introduced the same ratio of bases B(B = G/T/C) and S(S = G/C) as G/C-rich modules and same ratio of bases of T, K(K = G/T) and Y(Y = C/T) as T-rich modules. In this way, we got 6 series of design and we supplemented an extra design containing base-A. We inserted these different 30 bases closely between the TATA-box and the TSS sites of the promoters P_EXP1_ and P_GPD_, generating new promoters with complete architecture (Additional file 1: Figure S1). The constructed libraries were introduced respectively into two starting yeast strains (producing 1.2 mg/gDCW and 25 mg/gDCW of lycopene), getting LE (P_EXP1_ series in low lycopene-producing strain), HE, LG, HG (P_GPD_ series in high lycopene-producing strain) series of yeast colony libraries (Additional file 1: Figure S2).

**Table 1:** The sequence features of different core promoter libraries

Library no.	Sequence features of artificial 30 bases
1	T-rich 10 bases + T-rich 10 bases + T-rich 10 bases
2	T-rich 10 bases + T-rich 10 bases + C/G-rich 10 bases
3	T-rich 10 bases + G/C-rich 10 bases + T-rich 10 bases
4	G/C-rich 10 bases + T-rich 10 bases + T-rich 10 bases
5	G/C-rich 10 bases + T-rich 10 bases + G/C-rich 10 bases
6	G/C-rich 10 bases + G/C-rich 10 bases + G/C-rich 10 bases
7	T-rich 10 bases + T/A-rich 10 bases + T/A-rich 10 bases

### Artificial core promoter libraries tuned yeast colony-color spectrum

**Fig. 2:**
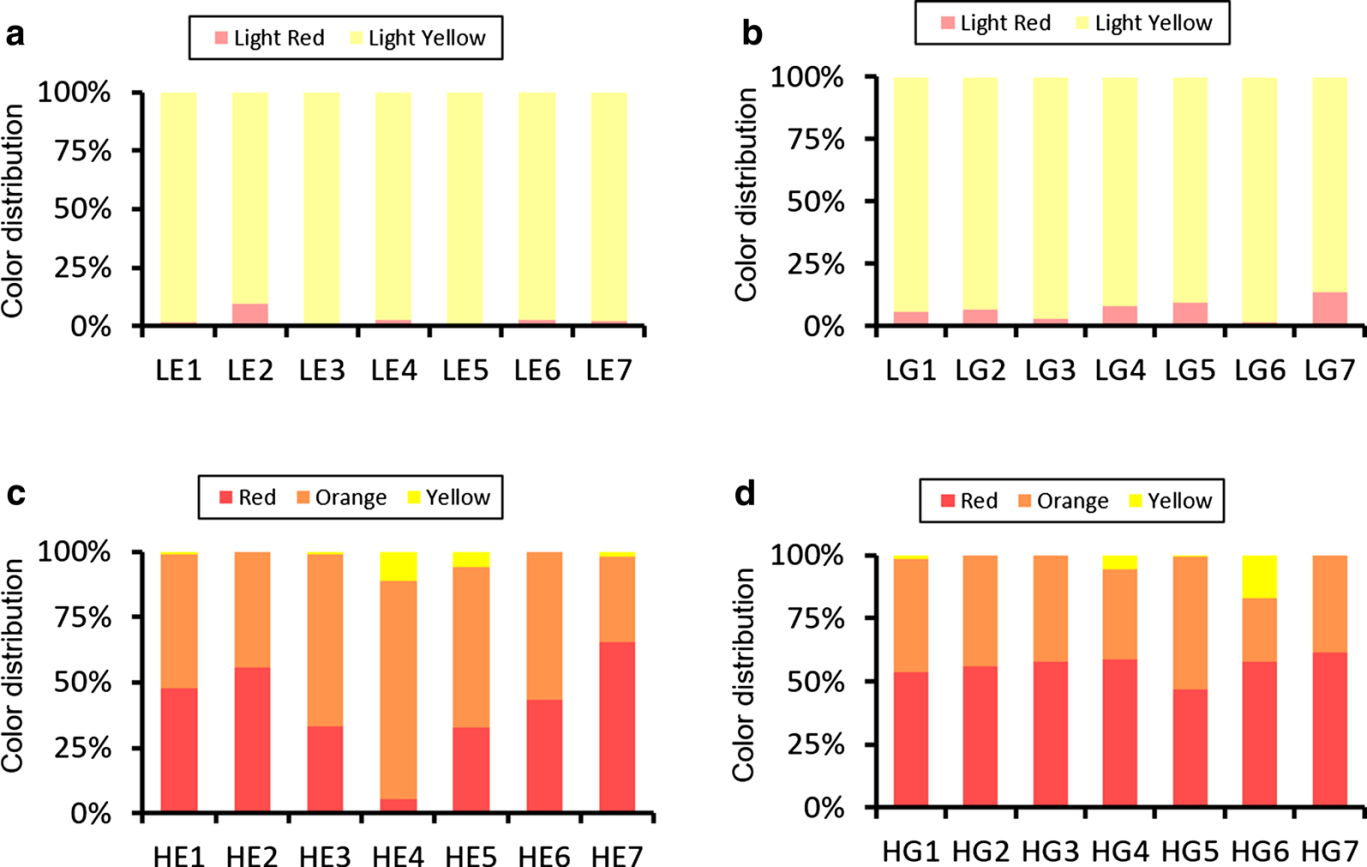
Colony-color spectrum represented the features of promoter libraries. The transformed yeast colonies presented distinguishable color spectrum. **a**, **b** The LE and LG series of libraries were constructed based on the low-lycopene-production strain. **c**, **d** The HE and HG series were based on the higher-lycopene-production strain

The transformed yeast colonies presented distinguishable colors of light red, light yellow, and red, yellow and orange (Fig. 2). In LE and LG libraries, the expression of CrtY transformed most of the light-red colonies into light-yellow colonies. However, the HE and HG libraries obviously presented orange colonies besides red ones and yellow ones. The LE libraries generally showed higher proportion of yellow colonies than LG libraries. Similarly, the HE libraries showed higher proportion of orange and yellow colonies than HG libraries. A possibility existed that the color was so light and hard to be judged as light red or light yellow. In the strains with 20-fold higher lycopene production, the flux of substrate was high enough and the variance of engineered promoters could be fully represented through colonies dyed with the colors of lycopene and carotene.

The combination of different upstream promoter regions and downstream 30 bases affected the whole promoter’s strength to different extents (Fig. 2). The P_EXP1_ with 30 random bases owned more tunable feature that made the color-spectrum differentiate among the colony libraries (5.5–65% red color colonies among HE libraries). Relatively, P_GPD_ was much more robust and all HG libraries remained around 53% red-color colonies. However, the intuitive color distribution did not mean the strength of P_GPD_ was harder to be enhanced. In order to get improved promoters, we further detected some typical orange and yellow colonies from both promoter libraries.

### Artificial core promoter libraries tuned CrtY expression and affected the synthesis of beta-carotene and lycopene

**Fig. 3:**
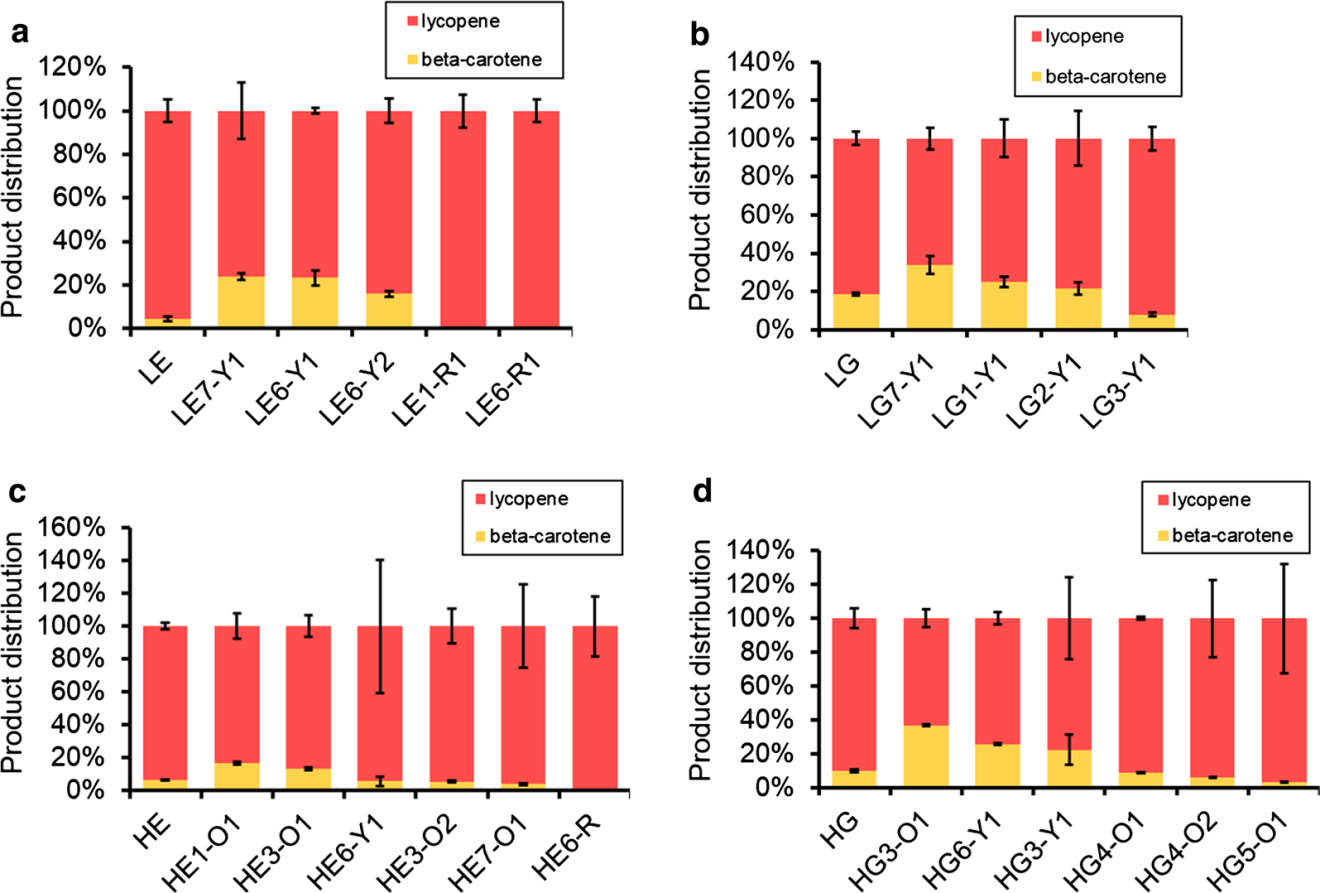
The different selected promoters correlated with distinguishable lycopene–carotene composition. The lycopene–carotene compositions of the selected strains were detected. The results of LE and LG series of strains were shown in **a**, **b** and the results of HE and HG series of strains were shown in **c**, **d**. An example of strain nomenclature: LE7-Y1, which indicates yellow colony 1 (-Y1) of the library No. 7 of the P_EXP1_ promoter (E7) in low lycopene producing strain (L)

Several colored colonies were picked up from the libraries on agar plates for measurement of their inner production of lycopene and beta-carotene (Fig. 3, Additional file 1: Figure S3). The strains picked from LE libraries showed an obviously improved transformation of lycopene to beta-carotene, compared with only 4.3% transformation of lycopene into beta-carotene in the strain with wild-type P_EXP1_. Strain LE7-Y1 and LE6-Y1 obtained highest transformation of lycopene–carotene as 23.8% and 23.3%, 5.5-fold and 5.4-fold of the value of the wild-type P_EXP1_. Only strain LG7-Y1 obviously performed better than the wild-type P_GPD_ and 33.9% of lycopene–carotene transformation was obtained compared with the 18.5% of the wild-type.

**Fig. 4:**
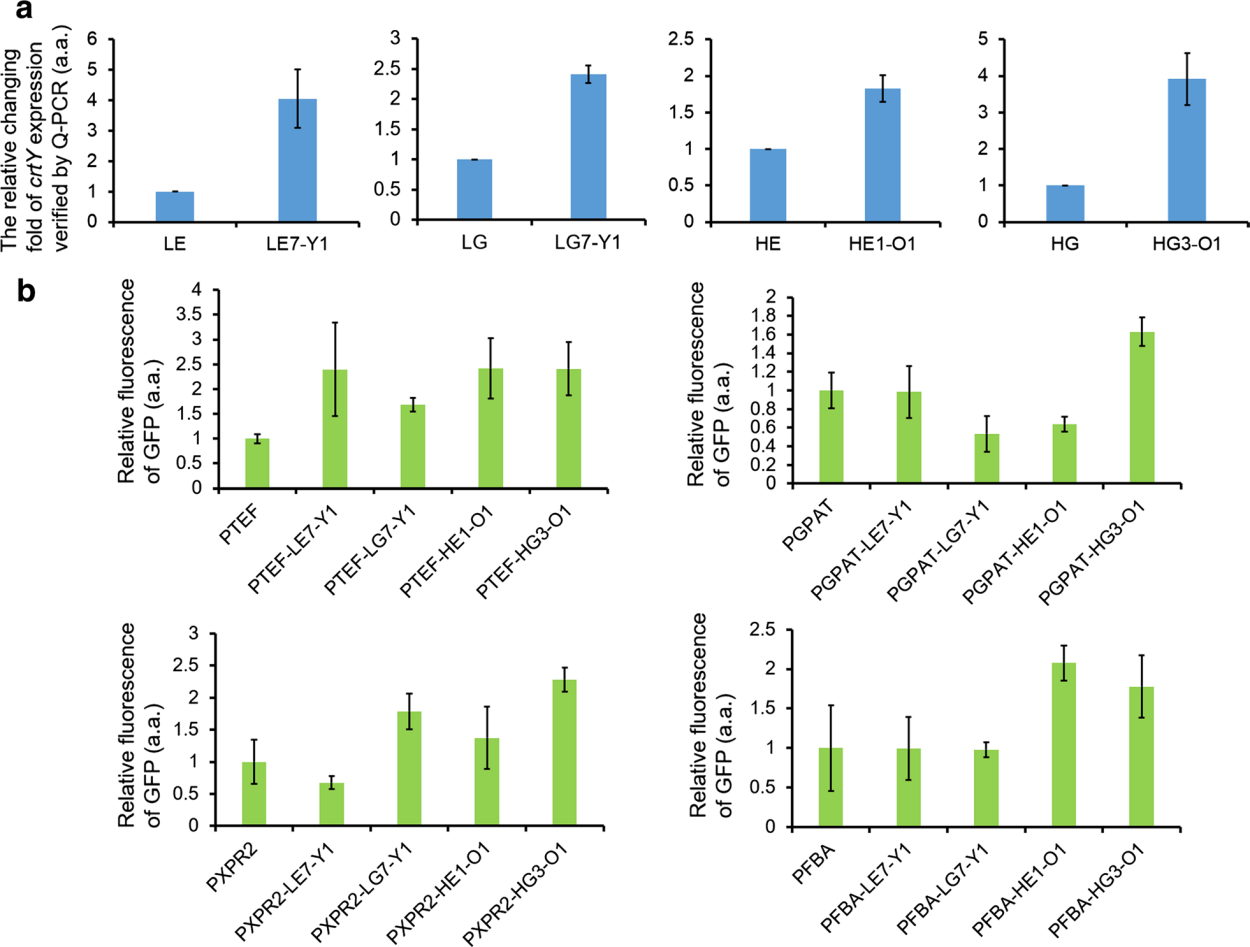
The Q-PCR verification of the enhanced promoters and the impacts of the artificial sequences combined with other natural promoters. **a** The relative changing fold of crtY transcription in the selected four champion strains (LE7-Y1, LG7-Y1, HE1-O1, HG3-O1) compared with their corresponding control strain were verified by Q-PCR. **b** The artificial sequences in the four champion strains were added to other promoters and the impacts on promoters’ strengths were detected in the form of relative fluorescence of GFP

As for the HE and HG libraries, large amounts of newly occurred orange colonies implied the increment of enzyme expression and metabolic product transformation (Fig. 3). Strain HE1-O1 and HE3-O1 got 16.6% and 13.0% transformation of lycopene–carotene, compared with the 6.2% of the wild-type. We selected more desirable promoters from HG libraries. Strain HG3-O1, HG6-Y1 and HG3-Y3 got separately 36.7%, 25.7% and 22.4% transformation of lycopene–carotene, compared with the 9.8% of the wild-type. A Q-PCR experiment showed that the transcriptional levels of *crtY* in the “champion strains” of LE7-O1, LG7-Y1, HE1-O1 and HG3-O1 were all enhanced by folds of 1.8 to 4, proving the improved expression of the enzyme (Fig. 4a). Strain HG3-O1 got a highest production of beta-carotene in this work as 7.4 mg/gDCW (Additional file 1: Figure S4). Different artificial promoters also affected total titers of lycopene and beta-carotene (Additional file 1: Figure S4). The champion strains of LE7-O1 and HG3-O1 got much higher titers than respective control strains, implying the pull of metabolic flux towards the synthesis of total carotenoids. The existence of other colorless intermediates in the pathway also influenced the detected titers (Additional file 1: Figure S2).

The results showed that some special artificial 30 bases performed better than the natural 55 bases of P_EXP1_ and 61 bases of P_GPD_. Certain new 30 bases enhanced the promoter’s strength but some others (HE6-R, LE1-R1, LE6-R1) disturbed the promoter’s action. The inactive ones were all selected from P_EXP1_ libraries, implying the strength of this promoter was more susceptible to the modification of core promoter region than P_GPD_. Finally, although some strains of HE and HG libraries got higher beta-carotene composition, the lycopene composition was still major, allowing further engineering and screening of stronger artificial promoters.

### Well performed promoters owned typical sequence features

Previous studies predicted that the natural yeast promoters with different levels of strength could be predicted according to their sequence features [25, 26]. For the regions directly upstream of TSS, the T was significantly enriched and A was depleted [25, 26]. The core promoters with high maximal promoter activity tend to be T rich upstream of the main TSS, and have lower G/C content around their main TSS [25]. Especially, the T-rich elements as TTTT, CTTT, TTC, CTTA and TCTA were high-frequent upstream of TSS in strong promoters. In our study, we endued these features to the artificial core promoters, although their lengths of 30 bases were much shorter than natural 55 bases and 61 bases.

We analyzed the sequence features of the selected promoters and found that the well-performed and bad-performed ones owned predictive sequence features (Additional file 1: Tables S2, S3). It was concluded that the well-performed promoters owned either higher %T or T-rich elements or both. The best LE7-Y1 got much higher %T (40%) than other LE promoters (less than 20%). The stronger LG7-Y1 and LG1-Y1 owned 2 and 4 T-rich elements but other LG promoters owned 1 or none. HE1-O1 owned both much higher %T (73.3%, other were around 16.7% to 53.3%) and more T-rich elements (5, other were only 1–2) than other HE promoters (Additional file 1: Table S3). The extraordinary HG4-O1 also got high %T and more T-rich elements, but its strength was lower than HG3-O1. The ladder contained more G and poly G than the former, implied that the detailed base orders also influenced promoter’s strength [25].

In addition, we tested whether the selected artificial promoters could enhance gene expression when inserted between the TATA-box and TSS site of other natural promoters (Fig. 4b, Additional file 1: Table S4). As for P_TEF_ and P_XPR2_, most newly constructed promoters got improved activities. However, for P_GPAT_, we only got an improved one when natural upstream sequence was combined with “HG3-O1” artificial sequence. Similarly, only “HE1-O1” and “HG3-O1” improved promoter’s strength. As the strength of natural P_TEF_, P_EXP1_ and P_GPD_ were much higher than other three natural promoters, our work was still proved effective to enhance the activities of strong promoters.

## Discussion

The modular features of yeast promoters offered space to design and synthesize artificial promoters that promoted high-efficient gene expression. However, major modules owned highly conservative base motifs and offer little space of redesign [15, 16]. Redden’s work selected several well-performed artificial upstream activating site (UAS) and core promoter elements from respective more than 10^6^ options [8]. The process of FACS, colony and sequencing analysis offered a standard procedure for promoter selection. In our work, the strength of redesigned promoter was correlated with the expression of a certain enzyme CrtY (Fig. 1). As a result, the yeast strains with different initial level of lycopene presented the transformation of lycopene into beta-carotene to differentiate extents (Additional file 1: Figure S2). Our work directly combined the demand of metabolic optimization in *Y. lipolytica* with promoter engineering, offering an approach of rapid screening of artificial promoters. The three partitions of 10-base T-rich or G/C-rich elements were joined together as the artificial 30 bases of core promoters of *Y. lipolytica* (Table 1).

The options of different promoter upstream sequences and different starting strains affected the variance of colony-color distribution, facilitating the screening of desirable engineered promoters. It was suggested that the higher metabolic flux could be an amplifier of the impacts of engineered promoters (Fig. 2). The HE and HG libraries showed obvious large amounts of orange colonies besides yellow colonies than the LE and LG libraries, implying the different ratios of lycopene and beta-carotene with much higher yields would obviously affect yeast phenotype to different extents. Different promoter’s upstream sequences also introduced different influences. The P_EXP1_ with 30 artificial bases were more susceptible than P_GPD_, as the red-color colonies of different HE libraries covered around 5.5–65% but all HG libraries remained 53% red-color colonies. P_GPD_ was more robust than P_EXP1_.

The different yields of lycopene and beta-carotene produced by the selected strains showed that not all the artificial promoters remained active (Fig. 3). Some red colonies like LE1-Y1, LE6-Y3 and HE6-R did not produce beta-carotene, implying the absence of the activity of CrtY. The selected best-performed artificial core promoters like LE7-Y1, LG7-Y1 and HG3-O1 obtained typical more T-rich elements or higher T-percentage than weak promoters (Additional file 1: Tables S2, S3). Portola’s recent work observed no meaningful correlation between nucleosome occupancy and promoter strength, not in accordance with previous opinion that AT rich sequences were associated with low nucleosome affinity and high promoter activity [27, 28]. Our work here found that the artificial strong promoters and natural strong ones both shared the common feature of T-rich elements and higher T-percentage. The conclusion was also verified when the best-performed artificial core promoters were combined with other upstream promoter sequences (Fig. 4).

The selected champion promoters in HE and HG libraries still did not transformed all the substrate lycopene into beta-carotene (Fig. 3, Additional file 1: Figure S4). This meant the starting high level of lycopene offered enough space for selection of improved promoters. The redesigned artificial promoters share different base orders and lengths from the traditional sequences. The de novo designed characteristic allows unique and orthogonal targeting of the promoter, which can be used as barcode. The unique barcode is beneficial to tuning and analysis of gene expression, targeting and recombination. Due to their diversity and independence of natural sequences, the artificial core promoters are valuable to the research of synthetic biology at pathway and genome level.

## Conclusions

In this study, we correlated the yeast artificial promoters with CrtY expression and the transformation of lycopene to beta-carotene which dyed yeast colonies with differentiate color spectrum. In this way, we selected some better-performed players and found that they owned higher %T and/or more T-rich elements, which was also typical features of natural stronger yeast promoters [25, 26]. In a similar way, a genomic scale of artificial barcode could be inserted into gene transcription, since the functional transcription regions had already been detected by RNA sequencing [29]. The de novo designed effective parts would facilitate the research of synthetic biology at the pathway level and genome level.

## Materials and methods

### Strains and media

All the *Y. lipolytica* strains used in this study were constructed from the wild-type strain ATCC 201249 (*MATA ura3*-*302 leu2*-*270 lys8*-*11 PEX17*-*HA*) [30]. *E. coli* strain DH5α (TransGen Biotech) was used for transformation of DNA materials. The YPD medium (20 g/L dextrose, 10 g/L yeast extract and 20 g/L peptone) and synthetic medium [0.67% yeast nitrogen base without amino acids, 2% glucose, and appropriate amino acid drop-out mix, lacking uracil (SC-Ura) and/or leucine (SC-Leu)] were respectively used for cultivation of the wild-type *Y. lipolytica* strain and the strains transformed with DNA. LB + Amp medium (10 g/L NaCl, 5 g/L yeast extract, and 10 g/L peptone, containing and 100 μg/mL ampicillin) was used to select transformed *E. coli* strains.

The two starting engineered strains (L-ini and H-ini, corresponding to the previous names of initial strain and A4AX1-25 strain, both for lycopene production) and the *crtY* transcription unit were chosen from our previous works [31, 32]. The *Y. lipolytica* strains producing lycopene and beta-carotene were inoculated in 4 mL of SC media in glass tubes for overnight cultivation at 28 °C and 250 rpm, and were then transferred into 50 mL of SC medium in three-parallel flasks with an initial OD_600_ of 0.1 and cultivated under the same condition. Twelve layers of aseptic gauze were used to seal the flask in order to ensure that the whole cell cultivation process was under aerobic condition. After 96 h, the cells were collected by centrifugation for measurement of cells’ dry weight and extraction of lycopene and beta-carotene.

### Construction of artificial core promoter libraries

All the artificial promoter libraries constructed in this study were upstream of *crtY* gene. The *crtY*-T_PEX18_ were cloned from the previously constructed unit with primers of P1-F and P1-R, and then were ligated in Yl-EcYl vector after digestion of BsaI [30, 31] (Additional file 1: Figure S1). The pre-located underlined sequence of GGAGACGTCGTCTCC were two back-to-back complementary BsmBI recognition sites. After BsmBI digestion, two sticky ends of AATG and TAAA would be generated for later DNA ligation (Additional file 1: Figure S1). After preparation of the control of inactivated *crtY* cassette, we firstly inserted the partial upstream sequences of P_EXP1_ and P_GPD_ respectively at the length of 701 bp and 617 bp. These two upstream parts were cloned with primers of P2-F, P2-R and P3-F, P3-R and digested with BsaI and then ligated upstream of *crtY*-T_PEX18_ (Additional file 1: Figure S1). In the downstream 299 bp of P_EXP1_ and 310 bp of P_GPD_, their respective core promoter sequences of 55 bp and 61 bp were replaced by libraries of 30 bp in design. The newly joined 274 bp and 279 bp in design for two promoters were split into eight oligonucleotides assisted by the DNA-design website (http://54.235.254.95/cgi-bin/gd/gdOliDes.cgi) The oligos were synthesized and assembled together with polymerase cycling assembly (PCA) as same as our previous method [6] (Additional file 1: Table S1). The assembled DNA segments were digested with BsaI and ligated between promoters’ upstream partial sequence and *crtY*-T_PEX18_. The *crtY* expression cassette was located on the single-copy plasmid of pLD-EcYl [31, 32]. According to the respectively designed 20 libraries of core promoter for both P_EXP1_ and P_GPD_, the ligated 40 mixed plasmids were respectively transformed into *E. coli* strain. After DNA transformation, all the *E. coli* colonies (about 500–1000) were washed from agar plates and the inner total plasmids were recovered and preserved at − 20 °C as constructed promoter libraries.

### Construction of yeast libraries with different colony-color spectrums

The constructed promoter libraries were transformed respectively into two starting engineered strains that produced lycopene, the low-lycopene-production strain and the high-lycopene-production strain [32] (Additional file 1: Figure S2). The classical LiAc/SS carrier DNA/PEG method was used for chemical transformation of *Y. lipolytica* cells. The total 360 μL transformation system contained 74 μL promoter libraries, 10 μL ssDNA, 240 μL PEG3350 (50%) and 36 μL LiAc (1 M). The transformed *Y. lipolytica* colonies were plated on Sc-Ura&/-Leu agar plates and cultivated at 28 °C for 3–5 days. Further, the number of colonies presenting distinguishable colors as light red, light yellow, red, yellow and orange would be counted (Fig. 1).

### Extraction of lycopene and beta-carotene from *Y. lipolytica* cells

The 1 mL of cells after 96 h cultivation were collected after centrifugation and 1 mL of ddH_2_O was added to wash the cells for twice. According to Xie’s method, 1 mL of 3 M HCl solution was added to resuspend yeas pellets and the mixture was boiled under boiling water for 3 min and then immediately cooled in ice bath for 5 min [33]. After centrifugation, the cell pellets were collected and washed by water for twice. 1 mL acetone containing 0.1% (w/v) BHT was added to the washed pellets and vortexed drastically for 15 min. After a final centrifugation, the upper layer of acetone phase was collected and filtered with organic filter membrane. All the centrifugation condition was 12,000 rpm for 5 min.

### Quantification of lycopene and beta-carotene

The extracted lycopene and beta-carotene contained in acetone phase was detected by lycopene and beta-carotene were quantitatively detected on a Waters 2695-2489 detector combined with an AscentisExpress C18 column (55 mm × 2.1 mm, 2.7 μm) with high performance liquid chromatography (HPLC). The sample volume was set to 10 μL, and the mobile phase was methanol:acetonitrile:dichloromethane as 40:9:1. The flow rate of mobile phase was 0.3 mL/min. The temperature of sample room was 22 °C and the column temperature was 25 °C. Detecting wavelength of lycopene was 470 nm and that of beta-carotene was 450 nm. Standards of lycopene and beta-carotene were purchased from SIGMA.

### Sequencing of the constructed artificial promoters

The selected yeast colonies were streaked on plates for purification. After that, the newly grown single colony was mixed with 50 μL of 20 mM NaOH and vortexed. The tubes were put in PCR equipment and an at least 3 times of cycling program of 99 °C and 4 °C were operated. 2–5 μL was used for PCR (total volume of 50 μL) and then the products were exhibited on gel electrophoresis. The extracted DNA products were sent for sequencing by Qingke Biotech and the results were summarized in Additional file 1: Table S2. The primers for sequencing were listed in Additional file 1: Table S1 as P_EXP1_-seqF, P_EXP1_-seqR, P_GPD_-seqF and P_GPD_-seqR.

## Supplementary information

**Additional file 1: Figure S1.** Illustration of the construction process of promoter libraries. **Figure S2.** The promoter libraries were tested in two initial *Y. lipolytica* strains. **Figure S3.** The selected strains were plotted again to show their colors. **Figure S4.** The production of lycopene and beta-carotene in the selected strains. **Table S1.** The primers used in this study. **Table S2.** Sequences of selected tested artificial promoters. **Table S3.** The sequence features of selected promoters. **Table S4.** The combination of artificial core promoters combined with natural promoter sequences.

## Data Availability

The datasets used and/or analyzed during the current study are available from the corresponding author on reasonable request.
