# Supplementary material for: Engineering yeast artificial core promoter with designated base motifs
Source: Microb Cell Fact. 2020 Feb 18;19:38. doi: 10.1186/s12934-020-01305-4 (PMC7026997; doi:10.1186/s12934-020-01305-4)
Supplement: Supplementary file 1 — Additional file 1: Figure S1. Illustration of the construction process of promoter libraries. Figure S2. The promoter libraries were tested in two initial Y. lipolytica strains. Figure S3. The selected strains were plotted again to show their colors. Figure S4. The production of lycopene and beta-carotene in the selected strains. Table S1. The primers used in this study. Table S2. Sequences of selected tested artificial promoters. Table S3. The sequence features of selected promoters. Table S4. The combination of artificial core promoters combined with natural promoter sequences. [file 12934_2020_1305_MOESM1_ESM.docx]

Additional file

**Engineering yeast artificial core promoter with designated base motifs**

Rui Liu^1,2^, Lanqing Liu^1,2^, Xia Li^1,2^, Duo Liu^1,2,^*, Yingjin Yuan^1,2^

1: Frontier Science Center for Synthetic Biology and Key Laboratory of Systems Bioengineering (Ministry of Education), School of Chemical Engineering and Technology, Tianjin University, Tianjin, 300350, P. R. China

2: SynBio Research Platform, Collaborative Innovation Center of Chemical Science and Engineering (Tianjin), Tianjin, 300072, P. R. China

*: Corresponding author: Duo Liu, E-mail: liuduo19870401@126.com

**List:**

**Figure S1.** Illustration of the construction process of promoter libraries.

**Figure S2.** The promoter libraries were tested in two initial *Y. lipolytica* strains.

**Figure S3.** The selected strains were plotted again to show their colors.

**Figure S4.** The production of lycopene and beta-carotene in the selected strains.

**Table S1.** The primers used in this study.

**Table S2.** Sequences of selected tested artificial promoters.

**Table S3.** The sequence features of selected promoters.

**Table S4.** The combination of artificial core promoters combined with natural promoter sequences.

**
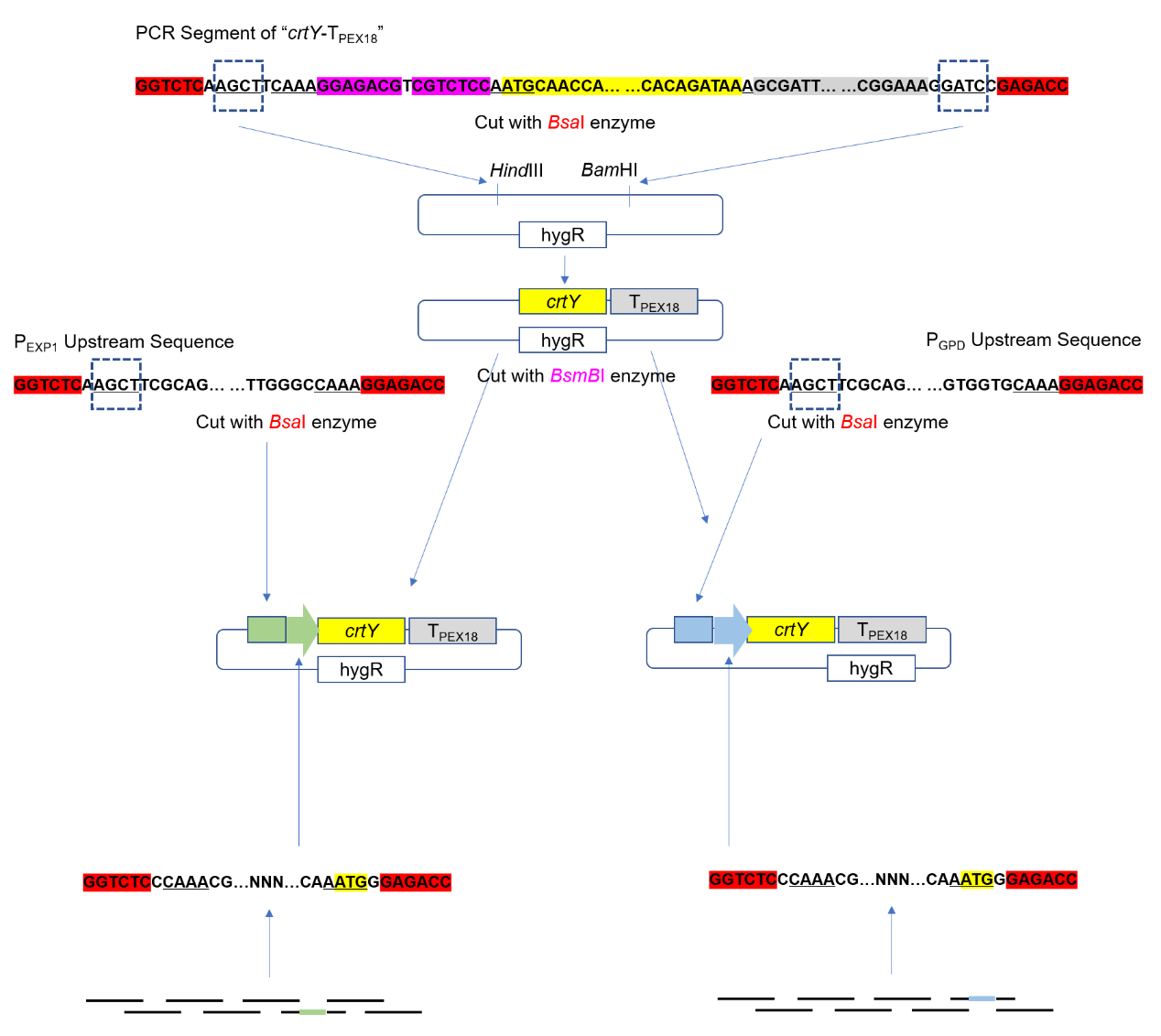
**

**Figure S1. Illustration of the construction process of promoter libraries.** The PCR segment of *crtY*-T_PEX18_ was cut with *Bsa*I enzyme and ligated into the plasmid pLD-EcYl that was digested with *Hind*III and *Bam*HI. The newly constructed blank TU of *crtY* was then cut with *Bsm*BI enzyme. Respective segments of P_EXP1_ and P_GPD_ upstream sequences after BsaI digestion were ligated before *crtY*.

**
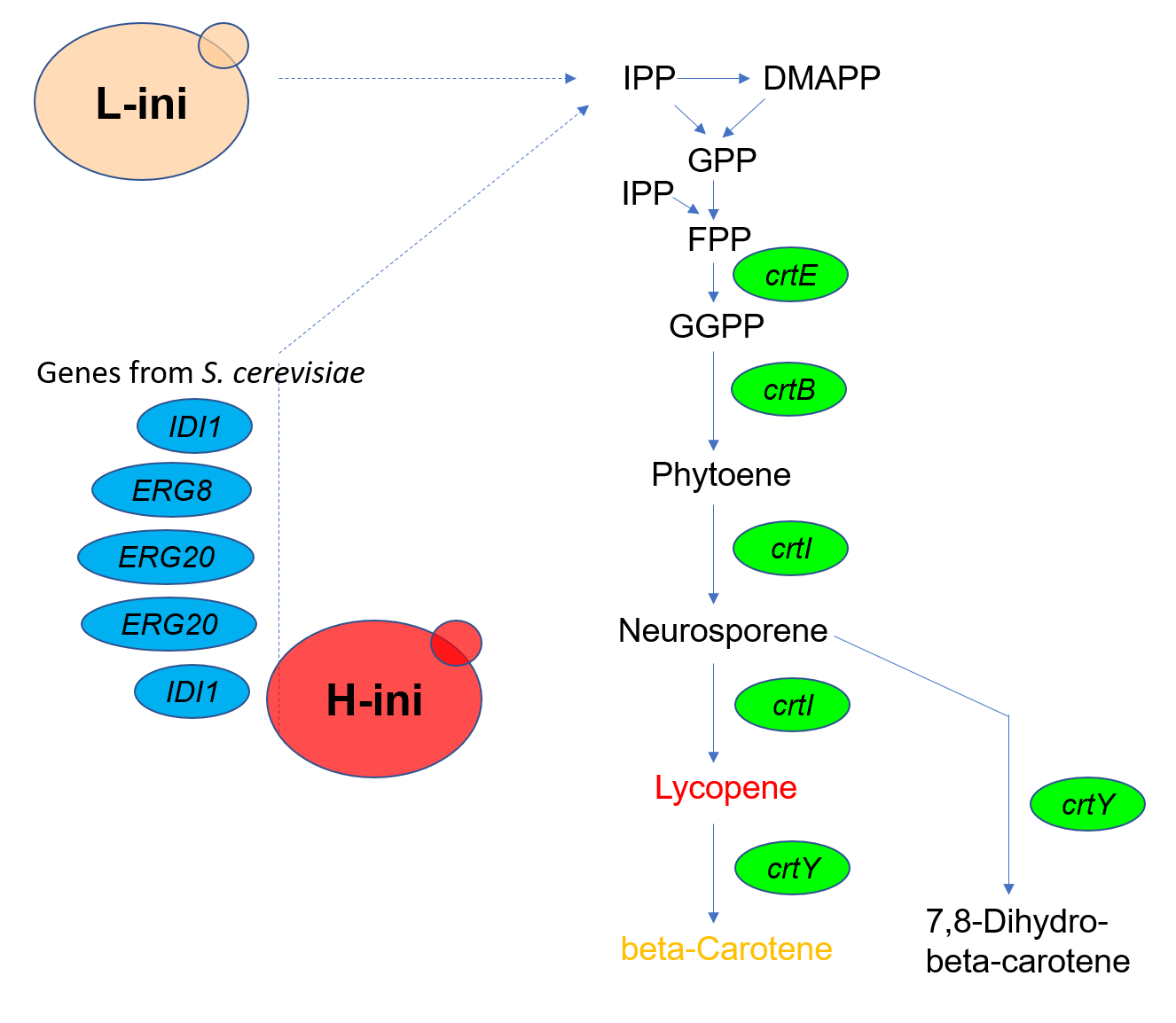
**

**Figure S2. The promoter libraries were tested in two initial *Y. lipolytica* strains.** The *crt* pathway (including *crtE*, *crtB*, *crtI* and *crtY*) was constructed in two strains. The strain only contained the pathway for lycopene synthesis and got a yield of 1.2 mg/gDCW production. By contrast, the strain contained other 5 transcriptional units for expression of two IDI1, two ERG20 and one ERG8 derived from *S. cerevisiae*. These genes made H-ini produce as high as 25 mg/gDCW lycopene.


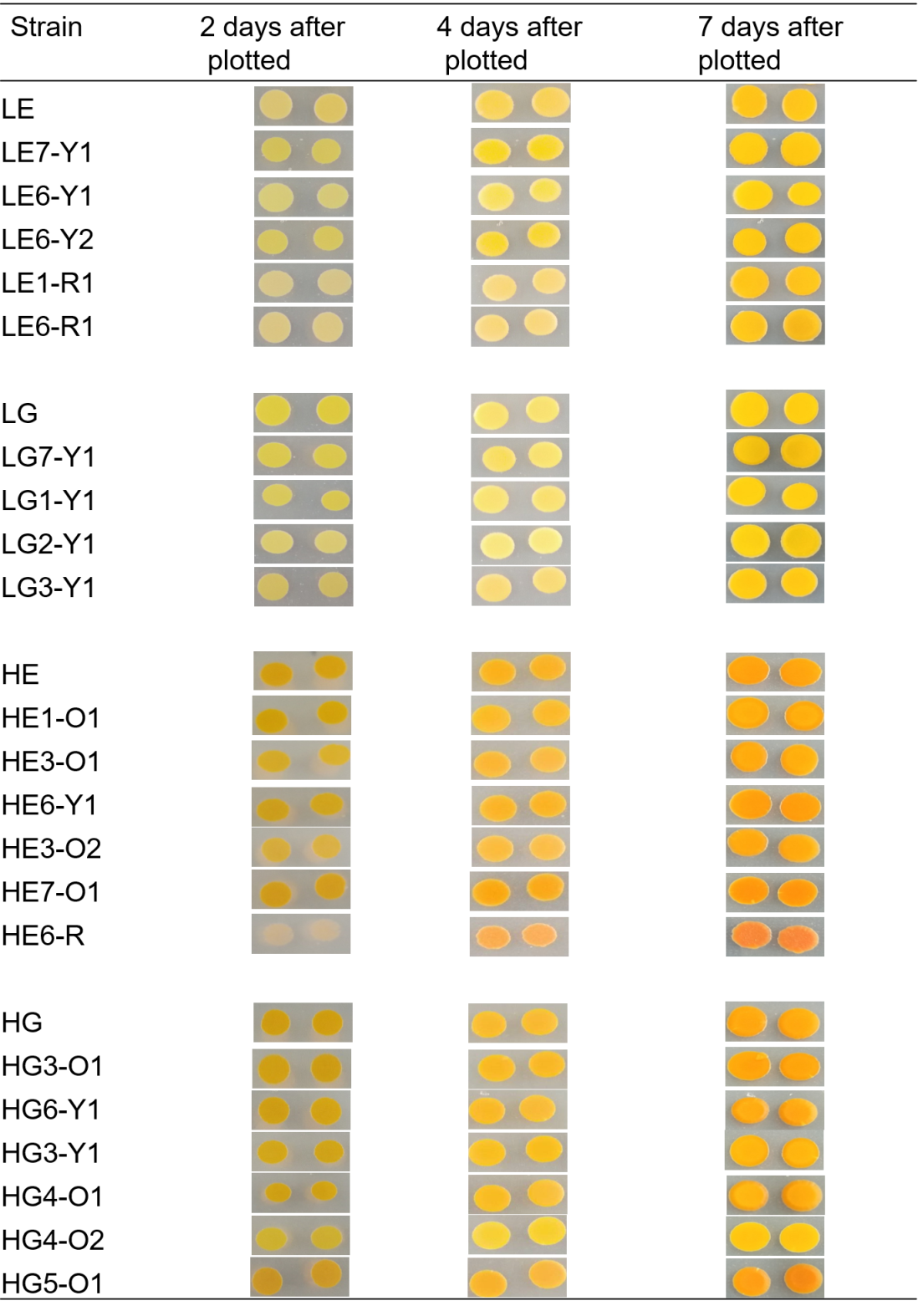


**Figure S3.** The selected strains were plotted again to show their colors. The strains were selected from the libraries of LE, LG, HE and HG. The different lycopene-carotene compositions in each strain were given in Figure 3 in the text and Figure S4.


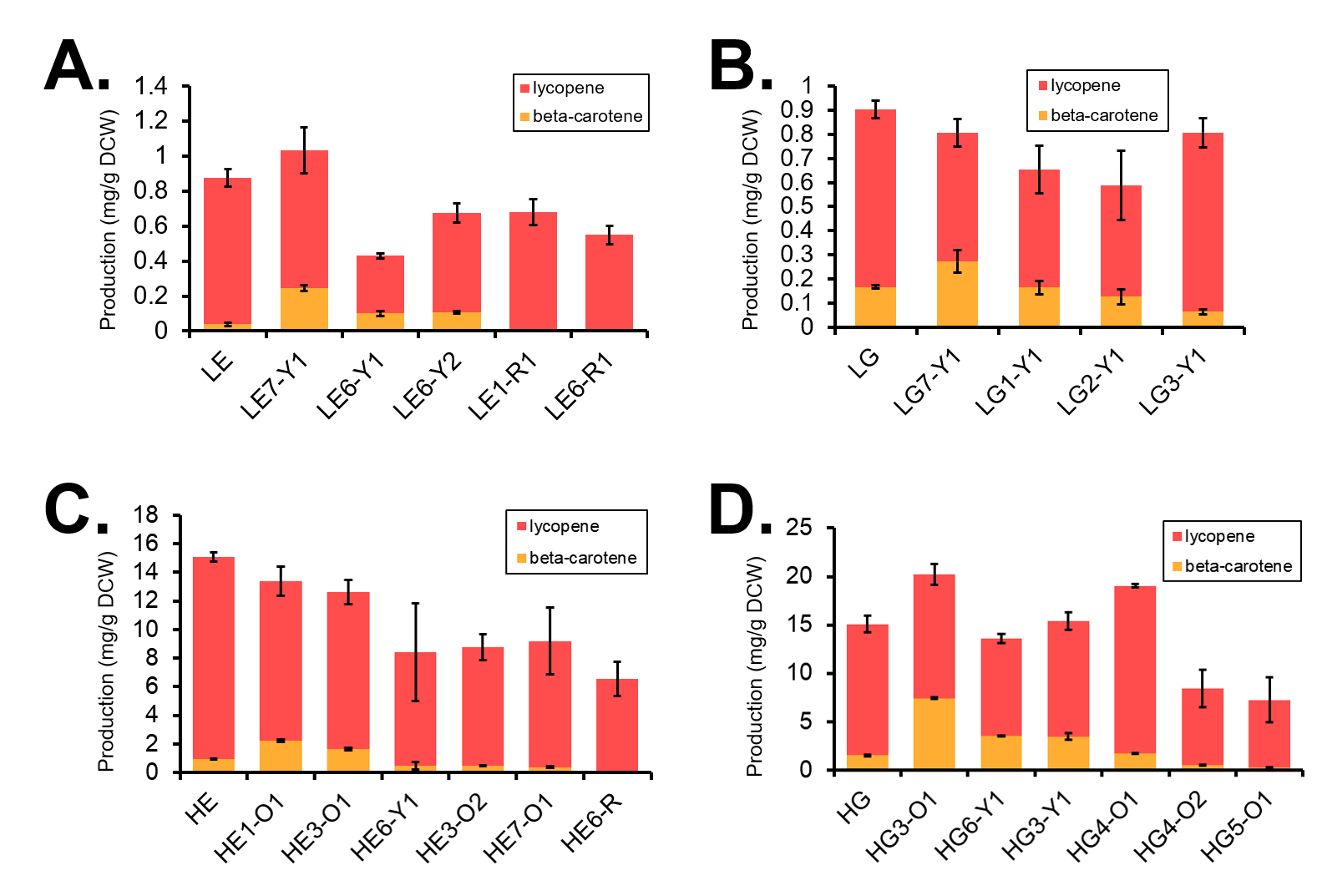


**Figure S4. The production of lycopene and beta-carotene in the selected strains.** The productions of lycopene and beta-carotene in each selected strain were detected. As shown, the total amounts were tuned to different extents when different artificial promoters were introduced to control the transcription of *crtY*.

**Table S1. The primers used in this study.**

| Primer | Sequence (5’ to 3’) |
| --- | --- |
| P1-F | GGTCTCAAGCTTCAAAGGAGACGTCGTCTCCAATGCAACCACACTACGACTTGATC |
| P1-R | GGTCTCGGATCCTTTCCGAGCTCGATGCTGGGAAG |
| P2-F | GGTCTCAAGCTTCGCAGGAGTTTGGCGCCCGTTTTTTCG |
| P2-R | GGTCTCCTTTGGCCCAAACATTTGTACGCGGT |
| P3-F | GGTCTCAAGCTTCGCAGTAGGATGTCCTGCACGGGTCT |
| P3-R | GGTCTCCTTTGCACCACTGCAGCCGCTTCT |
| PE-1 | GGTCTCCCAAAGTCTTGTCAGCCTTGCTTGCGAACCTAATTCCCAATTTTGTCACTTCG |
| PE-2 | GTTAGGGCTCGATCAATGGGGGTGCGAAGTGACAAAATTGGGAATTAGGTTC |
| PE-3 | CCCCATTGATCGAGCCCTAACCCCTGCCCATCAGGCAATCCAATTAAGCTCGCATTG |
| PE-4 | CGGGCAGGAGCCAAACTAAACAAGGCAGACAATGCGAGCTTAATTGGATTGCCT |
| PE-5 | TGTTTAGTTTGGCTCCTGCCCGTTTCGGCGTCCACTTGCACAAACACAAACAAGCATTATAT |
| PE-6-1 | GTGGTTGTTTGTGTTCTTGACTTTTGT….artificial bases….CTTATATATAATGCTTGTTTGTGTTTGTGCAAGTG |
| PE-7 | ACAAAAGTCAAGAACACAAACAACCACCCCAACCCCCTTACACACAAGACATA |
| PE-8 | GGTCTCCCATTTGCTGTAGATATGTCTTGTGTGTAAGGGGGTTG |
| PG-1 | GGTCTCCCAAACGGGGCGGAAACGGCGGGAAAAAGCCACGGGGGCACGAATTGA |
| PG-2 | GGGGCCGTGACTCGTCTCAAATTCGAGGGCGTGCCTCAATTCGTGCCCCCGTGG |
| PG-3 | TGAGACGAGTCACGGCCCCATTCGCCCGCGCAATGGCTCGCCAACGCCCGGTCT |
| PG-4 | ACACAAAGGTTTGGCTTGGGGTAACCTGATGTGGTGCAAAAGACCGGGCGTTGGCGAGC |
| PG-5 | TTACCCCAAGCCAAACCTTTGTGTTAAAAAGCTTAACATATTATACCGAACGTAGGTTTGGG |
| PG-6 | GTTGCCTTGGACAGACGGAGCAAGCCCGCCCAAACCTACGTTCGGTATAATATGT |
| PG-7 | CTCCGTCTGTCCAAGGCAACATTTATATAAG….artificial bases….AATTAAACACACATCAACAAATGGG |
| PG-8 | TGGATTTAGATAGTAAGTAGTGGGTCTCCCATTTGTTGATGTGTGTTT |
| P_EXP1_-seqF | CTGCCCATCAGGCAATCCAATTAA |
| P_EXP1_-seqR | ACCAACCAAGATCAAGTCGTAGT |
| P_GPD_-seqF | ATCAGGTTACCCCAAGCCAAA |
| P_GPD_-seqR | TGGTTGTTGTTGTTGCAATCTCAAA |

**Table S2. Sequences of selected tested artificial promoters.**

| Promoter name | Sequence (5’ to 3’) (artificial sequence was underlined) |
| --- | --- |
| LE7-Y1 | TATATAAGTGTGTTGGTGATTGAAACTTGCCTTGAGGTAC… |
| LE6-Y1 | TATATAAGGTTCCCGGGGTGGGGCCCCCGGGCCTCGGTAC… |
| LE6-Y2 | TATATAAGGTTCCCGGGGTGGGGCCCCCGGGCCTCGGTAC… |
| LE1-R1 | TATATAAGGTTCCCGGTTTGGGGCCCCCGGGCCTCCCCAC… |
| LE6-R1 | TATATAAGGTTCCCGGGGTGGGGCCCCCGGGCCTCGGTAC… |
| LG7-Y1 | TATATAAGTTCGCGTGTGCGCCGGCGGTTTCGGGGGCAAA… |
| LG1-Y1 | TATATAAGGGTGTTCGTGTGTTCGCTTCGTGTTCTGGGAA… |
| LG2-Y1 | TATATAAGTGGTTCGGCTGGTCCGGGCTTGTTGGCGGGAA… |
| LG3-Y1 | TATATAAGTGGTCCTCTTGCCCCCGGGTAGGTCCTGGGAA… |
| HE1-O1 | TATATAAGCGTTCTTGGCGGTCCTTTTCCGCCGGTTTCAC… |
| HE3-O1 | TATATAAGCGGCGTCTTTGCCTGCGGGGCGGTGTCCCTAC… |
| HE6-Y1 | TATATAAGGTTGGTCTGTTTCTGCTGTCTTGGTTTGCTAC… |
| HE3-O2 | TATATAAGGTTGGGTGTGTGTAAAACTGTTCCTGATTGAC… |
| HE7-O1 | TATATAAGGTTCCCGGGGTGGGGCCCCCGGGCCTCGGTAC… |
| HE6-R | TATATAAGGTTCCCGGGGTGGGGCCCCCGGGCCTCGGTAC… |
| HG3-O1 | TATATAAGGGGGGTCTTGGCTCGTTCTGTTTGGCCCTTAA… |
| HG6-Y1 | TATATAAGTTGGGGTTGGCCGTGCGCTCCTTGCCGGGGAA… |
| HG3-Y1 | TATATAAGTTGTGCTCGGGGTCGCTCTGTGTTGCGGCTAA… |
| HG4-O1 | TATATAAGTTGCGGCCCGTTTTCTTCTTCTGGTTTTGCAA… |
| HG4-O2 | TATATAAGTCTGCCCCTGCGTTTGGTGGGCCGGTCTGCAA… |
| HG5-O1 | TATATAAGGTTCGCCGCGGCTTTCTCGGTCTCCGGCGCAA… |

**Table S3. The calculated sequence features of selected promoters.**

| Promoter name | Strength level | %T | %G/C | No. of T-rich elements |
| --- | --- | --- | --- | --- |
| LE7-Y1 | *** | 40% | 43.3% | 0 |
| LE6-Y1 | *** | 16.7% | 83.3% | 1 |
| LE6-Y2 | *** | 16.7% | 83.3% | 1 |
| LE1-R1 | * | 20% | 80% | 0 |
| LE6-R1 | * | 16.7% | 83.3% | 1 |
| LG7-Y1 | *** | 23.3% | 76.7% | 2 |
| LG1-Y1 | *** | 43.3% | 56.7% | 4 |
| LG2-Y1 | ** | 30% | 70% | 1 |
| LG3-Y1 | * | 26.7% | 70% | 0 |
| HE1-O1 | *** | 73.3% | 26.7% | 5 |
| HE3-O1 | *** | 40% | 60% | 2 |
| HE6-Y1 | ** | 26.7% | 73.3% | 1 |
| HE3-O2 | ** | 53.3% | 46.7% | 1 |
| HE7-O1 | ** | 40% | 43.3% | 1 |
| HE6-R | * | 16.7% | 83.3% | 1 |
| HG3-O1 | *** | 40% | 60% | 2 |
| HG6-Y1 | *** | 26.7% | 73.3% | 0 |
| HG3-Y1 | *** | 36.7% | 63.3% | 0 |
| HG4-O1 | ** | 50% | 50% | 4 |
| HG4-O2 | * | 30% | 70% | 0 |
| HG5-O1 | * | 26.7% | 73.3% | 2 |

**Table S4.** The combination of artificial core promoters combined with natural promoter sequences.

| Natural promoter |  | Sequence of Artificial core promoters inserted between TATA-box and TSS of natural promoters |
| --- | --- | --- |
| P_EXP1_ |  | GGAGTTTGGCGCCCGTTTTTTCGAGCCCCACACGTTTCGGTGAGTATGAGCGGCGGCAGATTCGAGCGTTTCCGGTTTCCGCGGCTGGACGAGAGCCCATGATGGGGGCTCCCACCACCAGCAATCAGGGCCCTGATTACACACCCACCTGTAATGTCATGCTGTTCATCGTGGTTAATGCTGCTGTGTGCTGTGTGTGTGTGTTGTTTGGCGCTCATTGTTGCGTTATGCAGCGTACACCACAATATTGGAAGCTTATTAGCCTTTCTATTTTTTCGTTTGCAAGGCTTAACAACATTGCTGTGGAGAGGGATGGGGATATGGAGGCCGCTGGAGGGAGTCGGAGAGGCGTTTTGGAGCGGCTTGGCCTGGCGCCCAGCTCGCGAAACGCACCTAGGACCCTTTGGCACGCCGAAATGTGCCACTTTTCAGTCTAGTAACGCCTTACCTACGTCATTCCATGCATGCATGTTTGCGCCTTTTTTCCCTTGCCCTTGATCGCCACACAGTACAGTGCACTGTACAGTGGAGGTTTTGGGGGGGTCTTAGATGGGAGCTAAAAGCGGCCTAGCGGTACACTAGTGGGATTGTATGGAGTGGCATGGAGCCTAGGTGGAGCCTGACAGGACGCACGACCGGCTAGCCCGTGACAGACGATGGGTGGCTCCTGTTGTCCACCGCGTACAAATGTTTGGGCCAAAGTCTTGTCAGCCTTGCTTGCGAACCTAATTCCCAATTTTGTCACTTCGCACCCCCATTGATCGAGCCCTAACCCCTGCCCATCAGGCAATCCAATTAAGCTCGCATTGTCTGCCTTGTTTAGTTTGGCTCCTGCCCGTTTCGGCGTCCACTTGCACAAACACAAACAAGCATTA**TATATAAG**NNNNNNNNNNNNNNNNNNNNNNNNNNNNNNACAAAAGTCAAGAACACAAACAACCACCCCAACCCCCTTACACACAAGACATATCTACAGCA |
| P_GPD_ |  | GTAGGATGTCCTGCACGGGTCTTTTTGTGGGGTGTGGAGAAAGGGGTGCTTGGAGATGGAAGCCGGTAGAACCGGGCTGCTTGGGGGGATTTGGGGCCGCTGGGCTCCAAAGAGGGGTAGGCATTTCGTTGGGGTTACGTAATTGCGGCATTTGGGTCCTGCGCGCATGTCCCATTGGTCAGAATTAGTCCGGATAGGAGACTTATCAGCCAATCACAGCGCCGGATCCACCTGTAGGTTGGGTTGGGTGGGAGCACCCCTCCACAGAGTAGAGTCAAACAGCAGCAGCAACATGATAGTTGGGGGTGTGCGTGTTAAAGGAAAAAAAAAGAAGCTTGGGTTATATTCCCGCTCTATTTAGAGGTTGCGGGATAGACGCCGACGGAGGGCAATGGCGCCATGGAACCTTGCGGATATCGATACGCCGCGGCGGACTGCGTCCGAACCAGCTCCAGCAGCGTTTTTTCCGGGCCATTGAGCCGACTGCGACCCCGCCAACGTGTCTTGGCCCACGCACTCATGTCATGTTGGTGTTGGGAGGCCACTTTTTAAGTAGCACAAGGCACCTAGCTCGCAGCAAGGTGTCCGAACCAAAGAAGCGGCTGCAGTGGTGCAAACGGGGCGGAAACGGCGGGAAAAAGCCACGGGGGCACGAATTGAGGCACGCCCTCGAATTTGAGACGAGTCACGGCCCCATTCGCCCGCGCAATGGCTCGCCAACGCCCGGTCTTTTGCACCACATCAGGTTACCCCAAGCCAAACCTTTGTGTTAAAAAGCTTAACATATTATACCGAACGTAGGTTTGGGCGGGCTTGCTCCGTCTGTCCAAGGCAACATT**TATATAAG**NNNNNNNNNNNNNNNNNNNNNNNNNNNNNNAATTAAACACACATCAACA |
| P_TEF_ |  | AGAGACCGGGTTGGCGGCGCATTTGTGTCCCAAAAAACAGCCCCAATTGCCCCAATTGACCCCAAATTGACCCAGTAGCGGGCCCAACCCCGGCGAGAGCCCCCTTCTCCCCACATATCAAACCTCCCCCGGTTCCCACACTTGCCGTTAAGGGCGTAGGGTACTGCAGTCTGGAATCTACGCTTGTTCAGACTTTGTACTAGTTTCTTTGTCTGGCCATCCGGGTAACCCATGCCGGACGCAAAATAGACTACTGAAAATTTTTTTGCTTTGTGGTTGGGACTTTAGCCAAGGG**TATAAAAG**NNNNNNNNNNNNNNNNNNNNNNNNNNNNNNAGTATAAGAATCATTCAAA |
| P_GPAT_ |  | CAACTTTTCTTGTCGACCTGAGATACCGAGGTTGCGCAGGGGATCAACTTTTGTGTCTCAGAGGGACCCAAGTGCGTACGGAGAGTACAGTACATACTGTAGCTAACGGTAGCAGGCGAACTACTGGTACATACCTCCCCCGGAATATGTACAGGCATAATGCGTATCTGTGGGACATGTGGTCGTTGCGCCATTATGTAAGCAGCGTGTACTCCTCTGACTGTCCATATGGTTTGCTCCATCTCACCCTCATCGTTTTCATTGTTCACAGGCGGCCACAAAAAAACTGTCTTCTCTCCTTCTCTCTTCGCCTTAGTCTACTCGGACCAGTTTTAGTTTAGCTTGGCGCCACTGGATAAATGAGACCTCAGGCCTTGTGATGAGGAGGTCACTTATGAAGCATGTTAGGAGGTGCTTGTATGGATAGAGAAGCACCCAAAATAATAAGAATAATAATAAAACAGGGGGCGTTGTCATTTCATATCGTGTTTTCACCATCAATACACCTCCAAACAATGCCCTTCATGTGGCCAGCCCCAATATTGTCCTGTAGTTCAACTCTATGCAGCTCGTATCTTATTGAGCAAGTAAAACTCTGTCAGCCGATATTGCCCGACCCGCGACAAGGGTCAACAAGGTGGTGTAAGGCCTTCGCAGAAGTCAAAACTGTGCCAAACAAACATCTAGAGTCTCTTTGGTGTTTCTCGCATATATTTAATCGGCTGTCTTACGTATTTGGCCTCGGTACCGGACTAATTTCGGATCATCCCCAATACGCTTTTTCTTCGCAGCTGTCAACAGTGTCCATGATCTATCCACCTAAATGGGTCATATGAGGCGTATAATTTCGTGGTGCTGATAATAATTCCCATATATTTGACACAAAACTTCCCCCCCTAGACATACATCTCACAATCTCACTTCTTGTGCTTCTGTCACACATCTCCTCCAGCTGACTTCAACTCACACCTCTGCCCCAGTTGGTCTACAGCGGTATAAGGTTTCTCTGCATAGAGGTGCACCACTCCTCCCGATACTTGTTTGTGTGACTTGTGGGTCACGACA**TATATATC**NNNNNNNNNNNNNNNNNNNNNNNNNNNNNNAGCACAACAAAAACACGACACGCTAA |
| P_YAT1_ |  | ATAAGTTTGCAAAAAGATCGTATTATAGTTGGAGCAAGGGAGAAATGTAGAGTGTGAAAGACTCACTATGGTCCGGGCTTATCTCGACCAATAGCCAAAGTCTGGAGTTTCTGAGAGAAAAAGGCAAGATACGTATGTAACAAAGCGACGCATGGTACAATAATACCGGAGGCATGTATCATAGAGAGTTAGTGGTTCGATGATGGCACTGGTGCCTGGTATGACTTTATACGGCTGACTACATATTTGTCCTCAGACATACAATTACAGTCAAGCACTTACCCTTGGACATCTGTAGGTACCCCCCGGCCAAGACGATCTCAGCGTGTCGTATGTCGGATTGGCGTAGCTCCCTCGCTCGTCAATTGGCTCCCATCTACTTTCTTCTGCTTGGCTACACCCAGCATGTCTGCCATGGCTCGTTTTCGTGCCTTATCTATCCTCCCAGTATTACCAACTCTAAATGACATGATGTGATTGGGTCTACACTTTCATATCAGAGATAAGGAGTAGCACAGTTGCATAAAAAGCCCAACTCTAATCAGCTTCTTCCTTTCTTGTAATTAGTACAAAGGTGATTAGCGAAATCTGGAAGCTTAGTTGGCCCTAAAAAAATCAAAAAAAGCAAAAAACGAAAAACGAAAAACCACAGTTTTGAGAACAGGGAGGTAACGAAGGATCG**TATATATA**NNNNNNNNNNNNNNNNNNNNNNNNNNNNNNAACCAACCATTCTCACCACCCTAATTCACAA |
| P_XPR2_ |  | ACGCGTGGAGAGTTTGGGTTTTGGGTTACGTACGTAGAGCCGTTTGATAGATGGTACATCCACCGGCTAGCGGAACACAGTGTCAAGACAAGCCTGCAACACAGTCATAATATTTGCGATATTCAGGCGTATCAGGTACAATCTGAGGTGTCTCACAAGTGCCGTGCAGTCCCGCCCCCACTTGCTTCTCTTTGTGTGTAGTGTACGTACATTATCGAGACCGTTGTTCCCGCCCACCTCGATCCGGGGTCCTATGCATCCCTGAAACATTGATTGGAAATTAACATATGAGCTGCGTGCTTTTTGCATTCAAGGGCGCAGCTTATCTTGTATCCTTAATTACACATGACCTCTTGAGCGCCACGGTACATTCCTGGCGTCAGTTCGGTGGAGCGGACACTTTTCTCTCCTTTGTCTGACATGTTGGTTAAGTTGTAGTCCAGGGACACAAGGGGTTCCAACGGCAGTGGCAGCCTACCCCACGCTACCCACCACTGGCCCTGGTCTAACTTCGACGATCGGCATCAGGGTTCATGGATAGGCGGTGTGATTTACGATGTGATGGACAATGTTAGAGAGATCCCACTACTTGTAGTCAGGCCATCTTTTACGTACGCACTGTACCATGATGTCAATGGAGTATGATGAACCGACTTTGAGAGACTCACATCTGCACAACACCATGTTTCAGCGGAATCCGACTTCCAACCCAAACCCAAGCCCCTGTCAGATATCGTGAGAAGGCACGGCACCAACTAATGCACACACTCCACCTGTATTGCACCAAGATAATGAGGGCATCGTCTTGGCGCGTCTTGGCGAGAGCCGTGTTTCGTGACGCAATCAGAGCAGTTTCTGGATAGTATCTTGTCCAGAAACACGA**TATAAACC**NNNNNNNNNNNNNNNNNNNNNNNNNNNNNNCACTATCCAATCCTCCAATCCAACA |
| P_FBA_ |  | CAACAGTGTACGCAGTACTATAGAGGAACAATTGCCCCGGAGAAGACGGCCAGGCCGCCTAGATGACAAATTCAACAACTCACAGCTGACTTTCTGCCATTGCCACTAGGGGGGGGCCTTTTTATATGGCCAAGCCAAGCTCTCCACGTCGGTTGGGCTGCACCCAACAATAAATGGGTAGGGTTGCACCAACAAAGGGATGGGATGGGGGGTAGAAGATACGAGGATAACGGGGCTCAATGGCACAAATAAGAACGAATACTGCCATTAAGACTCGTGATCCAGCGACTGACACCATTGCATCATCTAAGGGCCTCAAAACTACCTCGGAACTGCTGCGCTGATCTGGACACCACAGAGGTTCCGAGCACTTTAGGTTGCACCAAATGTCCCACCAGGTGCAGGCAGAAAACGCTGGAACAGCGTGTACAGTTTGTCTTAGCAAAAAGTGAAGGCGCTGAGGTCGAGCAGGGTGGTGTGACTTGTTATAGCCTTTAGAGCTGCGAAAGCGCGTATGGATTTGGCTCATCAGGCCAGATTGAGGGTCTGTGGACACATGTCATGTTAGTGTACTTCAATCGCCCCCTGGATATAGCCCCGACAATAGGCCGTGGCCTCATTTTTTTGCCTTCCGCACATTTCCATTGCTCGGTACCCACACCTTGCTTCTCCTGCACTTGCCAACCTTAATACTGGTTTACATTGACCAACATCTTACAAGCGGGGGGCTTGTCTAGGG**TATATATA**NNNNNNNNNNNNNNNNNNNNNNNNNNNNNNAATGACACAATCCGAAAGTCGCTAGCAACACACACTCTCTACACAAACTAACCCAGCTCTT |
|  |  |  |
